# Antioxidant, Anti-Inflammatory, and Microbial-Modulating Activities of Nutraceuticals and Functional Foods

**DOI:** 10.1155/2017/7658617

**Published:** 2017-09-10

**Authors:** Ilaria Peluso, Thea Magrone, Débora Villaño Valencia, C.-Y. Oliver Chen, Maura Palmery

**Affiliations:** ^1^Research Centre for Food and Nutrition, Council for Agricultural Research and Economics (CREA-AN), Rome, Italy; ^2^Department of Basic Medical Sciences, Neuroscience and Sensory Organs, University of Bari, Bari, Italy; ^3^Universidad Católica San Antonio de Murcia (UCAM), Murcia, Spain; ^4^Antioxidants Research Laboratory, Jean Mayer USDA Human Nutrition Center on Aging, Tufts University, Boston, MA, USA; ^5^Department of Physiology and Pharmacology “V. Erspamer”, La Sapienza University of Rome, Rome, Italy

Oxidative stress is defined as the imbalance between reactive oxygen species (ROS) formation and enzymatic and nonenzymatic antioxidant defence favouring the former. Thus, enhanced oxidative stress occurs when ROS formation is increased by an array of exogenous and endogenous factors, for example, smoking, exposure to pollution, unhealthy diet, and chronic/low-grade inflammation, when the antioxidant defence capacity is compromised or both. Enhanced oxidative stress has been appreciated for its contribution to the pathogenesis of noncommunicable diseases (e.g., cardiovascular disease, metabolic syndrome, cancer, and brain disorders). Thus, to protect and prevent ROS-related pathogenesis, enhancing antioxidant defence through diets appears to be a viable strategy. From that, the antioxidant activity and the phytochemicals' content of foods and traditional medicine plants are an interesting topic of research. Antioxidant and antiproliferative activities, related to the content of phenolics, have been reported by M. N. Ombra et al. for twelve common bean (*Phaseolus vulgaris*) cultivars and by D. Zaluski et al. for the *Eleutherococcus senticosus* fruits intractum. I. Urquiaga et al. reported the antioxidant activity of berries' concentrate in humans after meal and discussed this result in terms of oxidative reactions that occur during digestion and the thermal processing of red meat.

In addition to acting as a ROS scavenger, polyphenols interact with many molecular targets after consumption. F. Giamogante et al. described the interaction between polyphenolic compounds and PDIA3, a molecule associated to different human pathologies. In particular, different flavonoids in virtue of their chemical backbone structure conformation can bind to PDIA3, thus preventing its interaction with DNA. ROS-induced DNA modifications can cause single-strand and double-strand breaks, leading to genotoxic effects. Thus, means that protect DNA from ROS and maintain DNA integrity have a chemopreventive potential. *Agaricus blazei* mushroom has been used in traditional medicine in the form of a medicinal extract for the prevention and treatment of cancers. L. Živkovic et al. found that this mushroom displayed antigenotoxic properties that were illustrated using Comet assay in human peripheral blood monocyte cells treated with H_2_O_2_ and acted as a strong •OH scavenger.

H. Wu et al. discussed on the inhibition of the high-mobility box 1-mediated pathway which could contribute to enhance anti-inflammatory and antifibrotic effects of resveratrol on diabetic cardiomyopathy disease.

In addition to attacking susceptible macromolecules, protein, DNA, and lipid, ROS modulate the expression of genes that are regulated by nuclear factor erythroid 2-related factor 2 (Nrf2) and nuclear factor-kappa B (NF-*κ*B). For example, Nrf2 stimulates the transcription of antioxidant enzymes through the antioxidant-responsive elements (ARE) and the activation of NF-*κ*B elicits the gene expression of inflammatory cytokines. Therefore, there is a concerted modulation of Nrf2 and NF-*κ*B in inflammation and oxidative stress. In this context, many nutraceuticals exert their anti-inflammatory and antioxidant effects through the inhibition of NF-*κ*B and the activation of Nrf2, respectively.

The organosulfur compound sulforaphane (SFN) has been evaluated by A. Dong et al., who observed that this compound upregulated Nrf2 expression as well as attenuated the inflammation process, by inhibiting NLRP3 and NF-*κ*B pathways, in acute pancreatitis, a pathology in which oxidative stress is closely related to tissue damage.

A. Gegotek et al. observed the effects of the flavonoid rutin in UV-irradiated fibroblasts, in which it reduced the proinflammatory response measured by TNF-*α* and NF-*κ*B levels; the oxidative response of xanthine and NADPH oxidases involved in the generation of superoxide radicals also diminished, linked with an increase in Nrf2 expression. E. Profumo et al. reported that the anti-inflammatory effect of the nutraceutical Dehydrozingerone is in part due to the inhibition of the NF-*κ*B pathway. On the other hand, the anti-inflammatory activity could be mediated also by other pathways. S. Qi et al. reported that myricitrin suppressed the production of free radicals and inflammatory cytokines and decreased the mRNA levels of nitric oxide synthase, the assembly of the components of the NADPH oxidase enzyme complex (gp91 phox and p47 phox), and the phosphorylation of Janus kinases (JAKs) and signal transducer and activator of transcription 1 (STAT1).

In order to overcome the problem of the low solubility and bioavailability of polyphenols, M. R. Lauro et al. developed and tested an eriocitrin/hydroxypropyl *β*-cyclodextrin inclusion complex as dietary flavonoid supplement to be employed in osteoarthritis.

In humans, also the subjects' characteristics influence the response to nutraceuticals. T. Magrone et al. reported that the peripheral blood mononuclear cells from obese and normal-weight subjects respond to the treatment with red grape polyphenols in vitro in a different manner, in particular, in terms of IL-2 and IL-21 production. A. Finamore et al. pointed out that the effects of *Spirulina* on cytokines and on lymphocytes' proliferation depend on age, gender, and body weight differences. Ageing and obesity are both associated with chronic low-grade inflammation, immune impairment, and intestinal dysbiosis. In this context, one of the most promising areas for the development of functional foods lies in modulation of the immune system by the use of probiotics, prebiotics, and synbiotics. Particularly, research examining the interactions occurring between probiotics, prebiotics, and nutraceuticals at the levels of the gut microbiota and of the gut-associated lymphoid tissue (GALT) is emerging. These interactions are relevant in the design of nutraceuticals and functional food for the prevention of noncommunicable diseases associated with dysbiosis, oxidative stress, and chronic low-grade inflammation.

P. Kleniewska and R. Pawliczak reported that a 7-week synbiotics (*Lactobacillus casei* + Inulin) supplementation improved oxidative stress markers (malondialdehyde, hydrogen peroxide, glutathione, and free sulfhydryl groups) in healthy subjects.

Medicinal botanicals contain a diverse range of substances exerting multifaceted bioactions potentially beneficial to human health. *Leonurus sibiricus L.* has been reported to possess cardioprotective, anticancer, analgesic, anti-inflammatory, and neuroprotective activities. Adding the list of these bioactions, P. Sitarek et al. examined the antibacterial, anti-inflammatory, antioxidant, and antiproliferative properties of essential oils in *Leonurus sibiricus L.* Using several in vitro platforms, they reported that essential oils, such as *β*-selinene, *β*-caryophyllene, and germacrene, contributed to the antibacterial, anti-inflammatory, antioxidant, and antiproliferative properties of *Leonurus sibiricus L*. These results support future studies to examine their therapeutic applications, as well as their use as food preservative. The same research group also applied the same methodologies to examine antimicrobial, anti-inflammatory, and antioxidant properties of a *Rhaponticum carthamoide* root. This medicinal plant has been traditionally used to treat overstrain and weakness. The study of E. Skala et al. showed that essential oils of *Rhaponticum carthamoide* root exhibited antimicrobial, anti-inflammatory, and antioxidant activities.

M. Remely et al. reported the beneficial effects of EGCG on gut microbiota, stressing out their anti-inflammatory, antioxidant, and epigenetic effects. M. Camps-Bossacoma et al. discussed on the potential tolerogenic role of cocoa diet on gut microbiota in orally sensitized mice. L. Dugo et al. examined the effect of cocoa polyphenolic extract on macrophage polarization from the proinflammatory M1 to the anti-inflammatory M2 state. They found in this vitro experiment that cocoa polyphenolic extract not only suppressed inflammation of macrophages in the M2 state but also changed macrophage metabolism by promoting oxidative pathways. However, the interpretation of the noted positive data must be taken with some cautions as cocoa polyphenols may not be present in the same chemical forms after absorption in the gastrointestinal tract. Further, future studies are warranted to identify pathways or molecular signals involved in the M1/M2 switch of macrophages.

In conclusion, this special issue highlights the importance of using different methodological approaches in order to clarify the mechanisms of the potential health effects of nutraceuticals.

